# Friction and Wear Reduction of Eccentric Journal Bearing Made of Sn-Based Babbitt for Ore Cone Crusher

**DOI:** 10.3390/ma9110950

**Published:** 2016-11-22

**Authors:** Auezhan Amanov, Byungmin Ahn, Moon Gu Lee, Yongho Jeon, Young-Sik Pyun

**Affiliations:** 1 Department of Mechanical Engineering, Sun Moon University, Asan 31460, Korea; pyoun@sunmoon.ac.kr; 2 Department of Materials Science, Engineering and Energy Systems Research, Ajou University, Suwon 16499, Korea; byungmin@ajou.ac.kr; 3 Department of Mechanical Engineering, Ajou University, Suwon 16499, Korea; moongulee@ajou.ac.kr (M.G.L.); princaps@ajou.ac.kr (Y.J.)

**Keywords:** Babbitt, friction, wear, ultrasonic nanocrystalline surface modification

## Abstract

An anti-friction Babbitt alloy-coated bearing made by a casting process is a journal bearing, which is used in an ore cone crusher eccentric. The main purpose of the Babbitt coated eccentric is to provide a low friction to support and guide a rotating shaft. Despite the fact that the Babbitt-coated eccentric offers a low friction coefficient and can be operated without a continuous supply of lubricant, it suffers from mining environments and short service life. In this study, an ultrasonic nanocrystalline surface modification (UNSM) technique was used to further reduce the friction coefficient, to increase the wear resistance, and to extend the service life of the Sn-based Babbitt metal. The friction and wear behavior of the Sn-based Babbitt metal was investigated using a block-on-ring tester under both dry and oil-lubricated conditions. The results of the experiments revealed that the friction and wear behavior of Sn-based Babbitt metal could be improved by the application of the UNSM technique. The friction and wear mechanisms of the specimens were explained and discussed in terms of changes in surface properties—microstructure, surface hardness, surface roughness, etc.

## 1. Introduction

The plain journal bearing (JB) is widely used in compressors, turbines, pumps, electric motors, electric generators, mining ore crushers, etc., because it provides a continuous sufficient lubrication supply to separate the moving parts by generating a hydrodynamic pressure between the journal and the shaft [1,2,3]. Improving the friction and wear behavior, load capacity, and extending the service life are the most important factors in designing JBs, in order to improve the efficiency and to reduce the number of replacements. An ore cone crusher is an application of a JB in the mining field, and the machine is designed to reduce large rocks into smaller rocks. In the ore cone crusher, the eccentric JB made of Sn-based Babbitt metal provides movement to the mantle, and then crushes the ore cone by squeezing. The ore cone crusher suffers from damage due to insufficient lubrication and severe wear, which mostly occurs on the surface of the eccentric JB. As a result, JBs have to be repaired or replaced frequently. The main purpose of the Babbitt metal eccentric is to provide a low friction to support and guide a rotating shaft of the ore cone crusher [4]. Although the Babbitt metal offers a low friction coefficient and can be operated reliably with a continuous pressurized supply of lubricant, it still suffers from damage due to continuous running time in mining and industrial environments. Ala-Kleme et al. have investigated the wear properties of an ore cone crusher [5]. In their experiment, the stones/rocks were crushed in compression in the cavity between the concave and the mantle. They have found that the microstructure of the specimen plays an important role in increasing the resistance to wear and its wear mechanisms. Another study on the correlation of material characteristics and cone crusher wear has been reported earlier [6]. It was found that the microstructure, hardness, etc. of materials have a significant influence on the performance and service life of ore cone crushers, where the wear of ore cone crushers has direct costs on service lifetime. Cleary et al. have also investigated the analysis of cone crusher performance with changes in material properties under different operating conditions [7]. They also confirmed the influence of the properties material on wear behavior. Moreover, Zhang et al. studied the friction and wear properties of Sn-based Babbitt bearing alloy with coating in dry and oil-lubricated conditions [8]. They compared the friction coefficient and wear of a Babbitt alloy with a coated one, where the coating reduced the friction coefficient and wear significantly in both dry and oil-lubricated conditions. According to the previous studies, it was found that, in order to increase the service lifetime of an ore cone crusher and to improve its performance, it is desirable to be able to modify the microstructure and surface of the materials. 

There are a number of methods and approaches to improve the efficiency of crushers, such as developing new materials, modifying the surface, improving the design and lubrication system, etc. Moreover, there are several types of surface modification approaches that can be applied to the parts of the crusher. For instance, surface texturing, which can create precise dimples on the surface. Those dimples can serve as a trap for wear debris, a reservoir for oil, and generate a hydrodynamic pressure, which in turn increases the oil film thickness, resulting in a reduction of the friction coefficient [9,10,11]. A multi-layer surface texture was applied to improve the tribological properties of the Babbitt alloy by creating grooves and dimples on the surface [12]. The dimples were created with different area density, depth, and groove width. The friction coefficient of the textured Babbitt alloy was found to be lower compared to that of the non-textured Babbitt alloy. The influence of thermal deformation treatment on the wear resistance of Babbitt alloy was previously investigated [13]. It was found that the creation of a nanostructure uniformly saturated with fine dispersed inclusions considerably increased the wear resistance. An alternative ultrasonic nanocrystalline surface modification (UNSM) technique—one of the widely used surface modification techniques—was employed. This technique increases the surface hardness, reduces the surface roughness, and refines the coarse grains into nano-sized grains [14], which improve the friction and wear behavior of materials. In addition, this technique is able to create micro-scale dimples on the surface of materials [15]. Further details of the UNSM technique and the dimensions of dimples can be found in our previous studies [16,17]. It was reported earlier that the UNSM technique can improve the frictional behavior of JBs made of bearing steel SAE 52100 [18]. According to the Stribeck curves at the boundary with mixed and hydrodynamic lubrication conditions, it was found that the friction coefficient of the UNSM-treated specimens was reduced by about 35%–50% compared to the untreated specimens. The reduction in the friction coefficient and the amount of wear in the UNSM-treated specimens was mainly attributed to the increase in hardness and the presence of dimples/grooves on the JB surface. The dimples/grooves serve as reservoirs for lubricant and provide a lubrication retaining effect in both starved and oil-lubricated conditions. They also generate a hydrodynamic bearing pressure, which uplifts of the contact specimen and creates a thin layer of oil in between, acting as a trap for wear debris in lubrication sliding contacts and reducing the third-body abrasion between sliding couples. Hence, the main objective of this study was to apply a newly developed UNSM technique for Sn-based Babbitt alloy to improve the friction and wear behavior of eccentric JBs for ore cone crushers.

## 2. Materials and Methods 

Babbitt alloys usually consist of a soft matrix and reinforcing phase inclusions. The α-phase (solid solution of antimony and copper in tin) is the matrix, and it provides good conformability and a special surface micro-relief, which improves the supply of oil by friction sites and heat extraction. Solid phase inclusions (β-phase, Sn and Sb) ensure a high level of wear resistance [19,20]. In this study, Sn-based Babbitt block specimens with dimensions of 40 × 10 × 10 mm^3^ and tool steel H13 eccentric ring specimens with a diameter of 30 mm and a length of 100 mm were used (Belmont, Brooklyn, NY, USA). The chemical composition of the Sn-based Babbitt specimens is listed in Table 1. 

The alloy contains Zn, which increases hardness, but deteriorates frictional behavior. It also contains around 5% Cu, which weakens the strength, but increases the melting point. The specimens were first ground to flatness and then mirror polished down to a particle size of 1 µm using an alumina suspension. Afterwards, the polished specimens were subjected to UNSM treatment under the optimized parameters listed in Table 2. 

**Figure 1:**
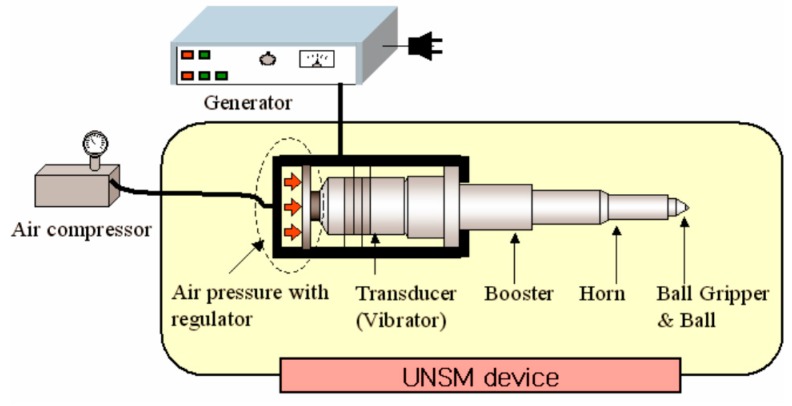
A schematic view of a UNSM device.

**Figure 2:**
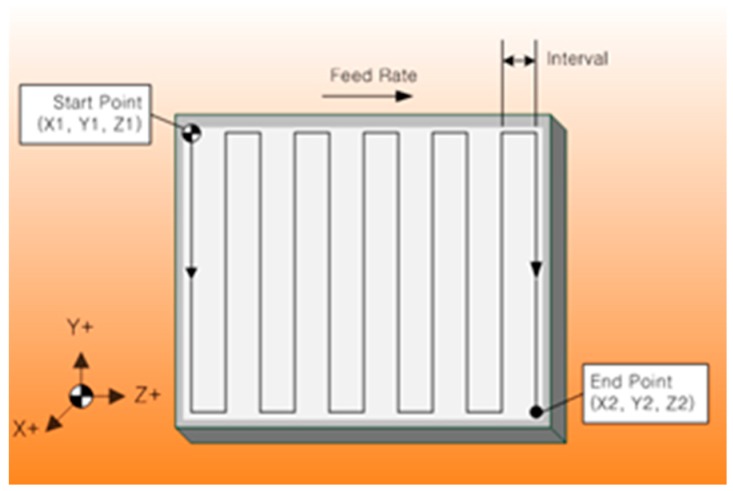
Diagram of fundamental parameters of the UNSM treatment.

The UNSM technique is a patented material strengthening technology. The UNSM technique strikes the specimen surface up to 20,000 times per second with a WC or a Si_3_N_4_ tip with a diameter of 2.38 mm, which modifies the coarse grains into nano-sized grains until a certain depth from the top surface. The schematic view of an in-house built UNSM technique is shown in Figure 1. In this UNSM treatment process, not only the static load (*P_st_*), but also the dynamic load (*P_dy_* = *P* sin 2π*ft*) are exerted to a surface, where *P* is the load, *f* is the frequency and *t* is the time. A generator and piezoelectric transducer (shown in Figure 1) emit ultrasonic waves at 20 kHz. The waves are amplified when they travel through an acoustic booster. The dimension of the vibrating part (which contacts the surface) allows vibration amplitudes of 10–100 µm to be attained. A homogenous treatment is obtained on the treated surface. The principle of UNSM is based on the instrumental conversion of harmonic oscillations of an acoustically-tuned body into resonant impulses of ultrasonic frequency. The acoustically-tuned body is brought to resonance by energizing an ultrasonic transducer. The UNSM technology device can be installed on any numerical control (NC) and/or computer numerical controlled (CNC) machines in different positions. In this study, the UNSM treatment process was performed on a standard NC lathe, which is retrofitted with a piezoelectric-actuated tool positioning stage. A diagram of the fundamental parameters of the UNSM treatment is depicted in Figure 2. More details of the UNSM treatment parameters and settings have been well described in previous publications [16,17].

**Figure 3:**
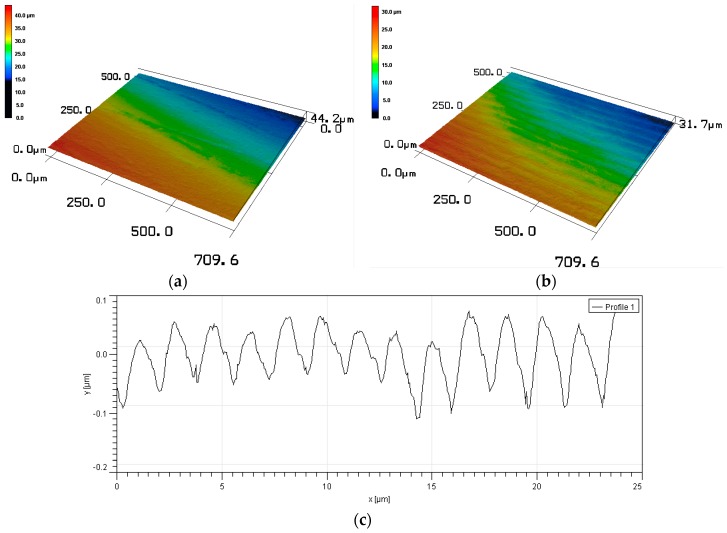

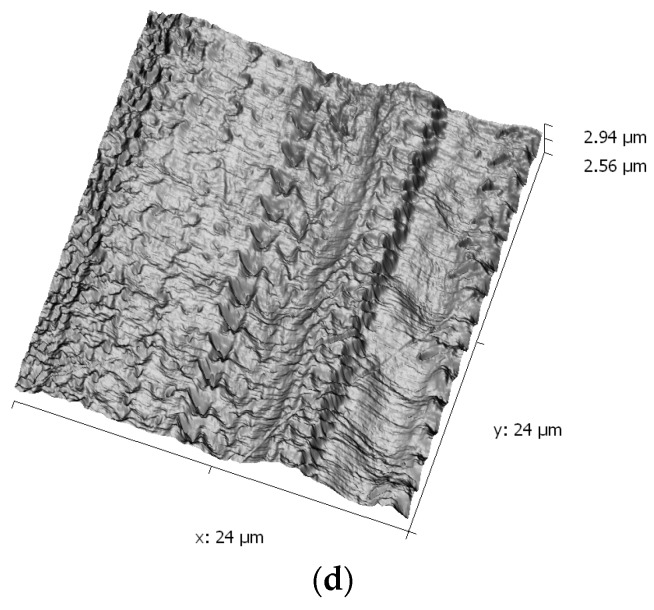
3D laser microscopic images of the (**a**) untreated and (**b**) UNSM-treated Babbitt specimens. Atomic force microscopy (AFM) images of the UNSM-treated Babbitt specimen showing (**c**) the wavy grooves and (**d**) dimples on the surface.

The microstructure of the untreated and UNSM-treated specimens is shown in Figure 3a,b, respectively. It is obvious that the untreated specimen has some porosities and defects on the surface, which are the results of casting technology. The microstructure of the Babbitt specimen was modified by UNSM technique, where the presence of wavy grooves on the surface was observed as shown in Figure 3b. Atomic force microscopy (AFM, Seiko Instruments SPA400, Chiba, Japan) was employed to confirm the presence of wavy grooves and dimples on the surface after UNSM treatment. The cross-sectional profile of the corrugated surface is shown in Figure 3c. It can be seen that the UNSM treatment can create a surface with continuous grooves. Additionally, the micro-dimples can be produced by UNSM treatment on the surface of the specimen. Figure 3d shows the 3D-AFM image of the UNSM-treated Babbitt specimen. It is obvious that a huge number of dimples can be observed after UNSM treatment. It has been previously reported that the friction and wear of materials can be controlled by the presence of dimples on the surface [11,14].

**Figure 4:**
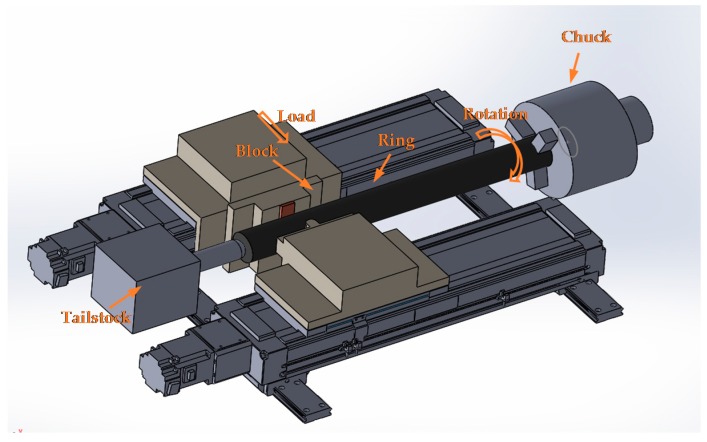
A schematic view of a block-on-ring friction tester used in this study.

In order to simulate similar conditions with the eccentric JB of a cone ore crusher, an in-house built block-on-ring (ASTM G77 standard) JB friction tester was used to simulate the friction and wear behavior of the specimens at an applied load of 400 N and a spindle speed of 100 rpm for an hour at room temperature in both dry and oil-lubricated conditions. A schematic view of a block-on-ring friction tester, which was built in-house, is shown in Figure 4. A commercial ISO VG 32 Standard SYNTHDRO 32AW oil (SHL Lubricants, Seoul, Korea) was used as a lubricant. The friction and wear tests were replicated at least three times in order to have a statistical significance of results. The wear loss of the specimens was measured using a precision balance ALC-310.3 with an accuracy of milligrams (Midland scales, West Midlands, UK). 

The surface hardness as a function of the depth from the top surface along with indentations and average surface roughness of the untreated and UNSM-treated Babbitt specimens were measured using a micro-Vickers hardness tester (MVK-E3, Mitutoyo, Chiba, Japan)at a load of 50 gf and a dwell time of 10 s, and a two-dimensional surface profilometer (SJ-210, Mitutoyo), respectively. The top surface nano-hardness of the specimens was measured using a nanoindentation technique (CSM Instruments, Peseux, Switzerland). The images and dimple profiles were obtained using a three-dimensional laser scanning microscope (LSM, VK-X100 series, KEYENCE, Shinagawa, Japan). The wear tracks of the specimens were observed using a scanning electron microscope (SEM, SNE-3000M, SEC Co., Ltd., Suwon, Korea) in order to investigate the wear mechanisms.

## 3. Results and Discussion

**Figure 5:**
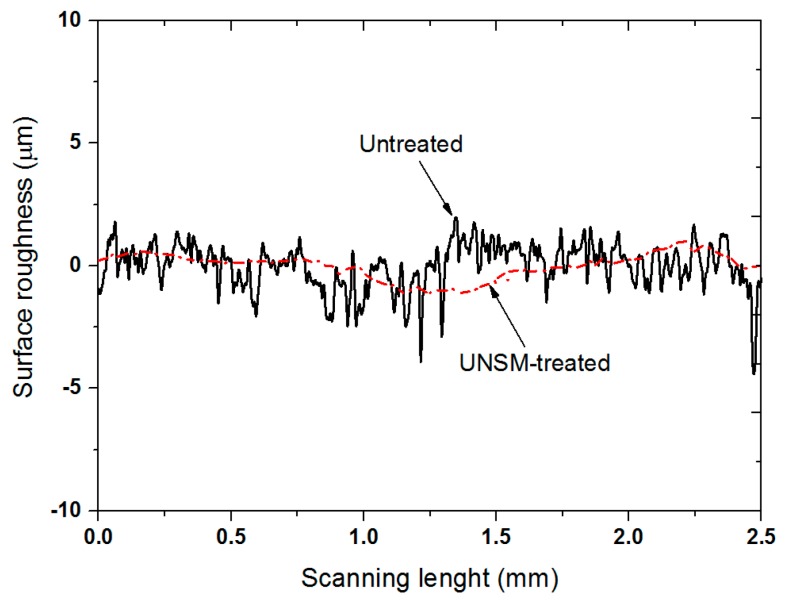
Comparison in surface roughness for the untreated and UNSM-treated Babbitt specimens. The average surface roughness (*Ra*) of the untreated and UNSM-treated specimens was about 0.26 and 0.14 µm, respectively.

Figure 5 shows the comparison of surface roughness for the untreated and UNSM-treated specimens. It can be seen that the average surface roughness (*Ra*) of the specimens was reduced significantly after UNSM treatment, which was found to be 0.26 and 0.14 µm for the untreated and UNSM-treated specimens, respectively. The reduction in surface roughness plays an important role in improving the friction and wear behavior of materials [21]. Eklund has studied the roughness effect on the friction and wear of lubricated plain bearings made of bearing materials such as copper, aluminum, and Babbitt alloys using a block-on-ring configuration [22]. It was found that the surface roughness of soft materials significantly affects the friction and wear behavior, where the friction and wear behavior improved with a reduction in the surface roughness. 

**Figure 6:**
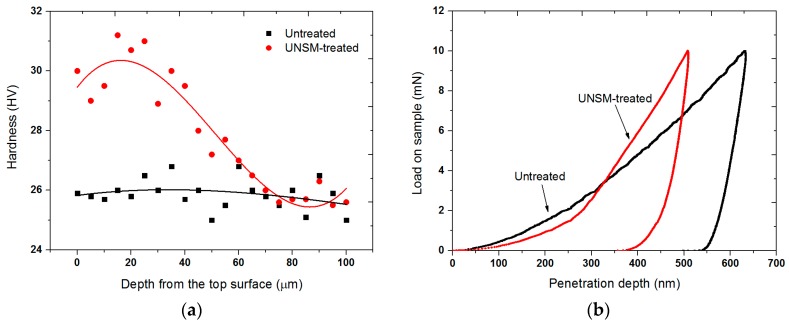
(**a**) Comparison in surface hardness with respect to depth from the top surface for the untreated and UNSM-treated Babbitt specimens: the surface hardness was about 25 and 31 HV for the untreated and UNSM-treated specimens, respectively; (**b**) Typical *P*-*h* curves of the untreated and UNSM-treated specimens obtained from nano-indentation.

**Figure 7:**
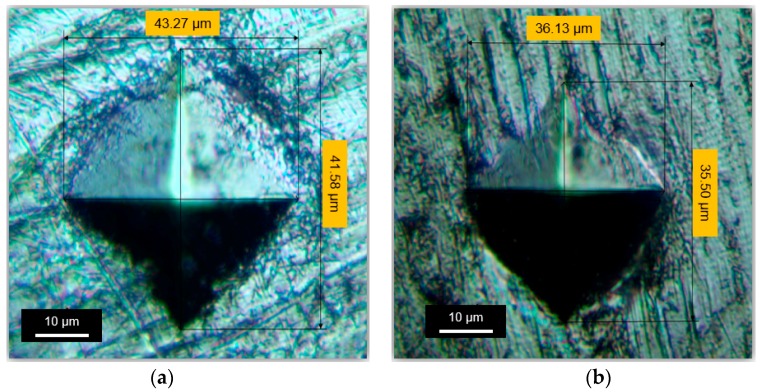
Comparison of indentations created on the surface of the (**a**) untreated and (**b**) UNSM-treated Babbitt specimens.

Figure 6 presents a comparison in surface hardness with respect to depth from the top surface for the untreated and UNSM-treated specimens. It was observed that the UNSM treatment increased the hardness of a Babbitt alloy by about 18% at the top surface, as shown in Figure 6a. However, the effective hardened layer with a thickness of about 75 µm decreased gradually with increasing depth. The top surface nano-hardness of the specimens was also measured. It was found from the typical *P*-*h* curve that the hardness of the UNSM-treated specimen at the top surface was found to be higher compared to that of the untreated specimen. Figure 6b also shows that the penetration depth was found to be about 635 and 502 nm for the untreated and UNSM-treated specimens, respectively. From the typical load-displacement curve obtained from the nanoindentation tests (see Figure 6b), the hardness and Young’s modulus of the specimens were calculated based on the method described by Oliver and Pharr. The top surface nano-hardness of the untreated and UNSM-treated specimens was about 135 and 167 MPa, while the Young’s modulus was 18.2 and 25.9 GPa, respectively. The increase in hardness of the Babbitt alloy can be explained by the Hall–Petch expression [23]. Hardness is the material quality that can determine the wear resistance to plastic deformation. Figure 7 shows the indentations created on the surface of the untreated and UNSM-treated specimens after the hardness test. It was observed that the diagonal length of the indentation created on the untreated specimen was longer compared to that of the UNSM-treated specimen. In the case of indentation, the smaller the indentation, the harder the material; confirming the increase in the hardness of the UNSM-treated specimen. Leszczynska-Madej and Madej have reported on the mechanism of hardness increase in Babbitt alloys [24]. They found that the increase in hardness of Babbitt alloys may be attributed to the presence of a hard SnSb phase in the form of squares in the microstructure and the occurrence of fine precipitates uniformly distributed over the whole Sn matrix. Hence, it is believed that the presence of hard phases formed after UNSM treatment could be the mechanism of hardness increase.

**Figure 8:**
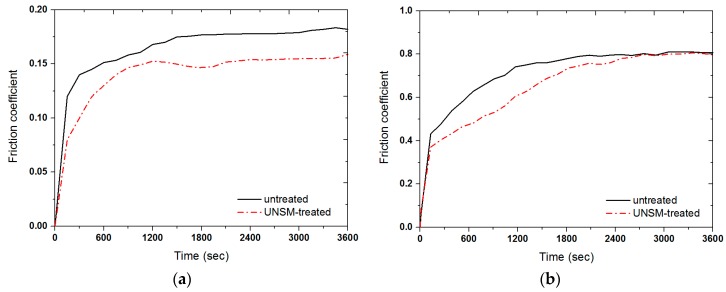
Variation in friction coefficient of the untreated and UNSM-treated Babbitt specimens in (**a**) dry and (**b**) oil-lubricated conditions, respectively.

Figure 8a,b shows the variation in the friction coefficient for the untreated and UNSM-treated specimens under dry and oil-lubricated conditions, respectively. It was observed that the UNSM-treated specimens exhibit a lower friction coefficient than the untreated specimens in both dry and oil-lubricated conditions. The friction increased gradually at the onset of the sliding, which may be attributed to the removal of adsorbed surface impurities and the deformation of contact asperities at the interface. The friction coefficient stabilized to steady-state values of 0.176 and 0.148, as well as 0.81 and 0.79, respectively, for the untreated and UNSM-treated specimens under dry and oil-lubricated conditions. Attainment of the steady-state in a short period of time by the UNSM-treated specimen under dry conditions, with a friction coefficient value of about 0.15, may be attributed to the low surface roughness, high surface hardness, and the presence of grooves on the surface produced by the UNSM technique. The friction coefficient of ingot and bearing Babbitt alloys has been compared under dry conditions in a previous study [24]. It was found that the bearing Babbitt—which had a higher hardness than that of the ingot Babbitt—exhibited a higher friction coefficient after a sliding distance of 1000 m. The reason for the high friction was explained by the fact that the hard phases on the wear surface during sliding were crushed and pulled out from the matrix and acted as hard abrasive particles that caused an increase in the friction coefficient. However, in contrast, the UNSM-treated Babbitt alloy showed a lower friction coefficient than that of the untreated Babbitt alloy under both dry and oil-lubricated conditions, as shown in Figure 8.

**Figure 9:**
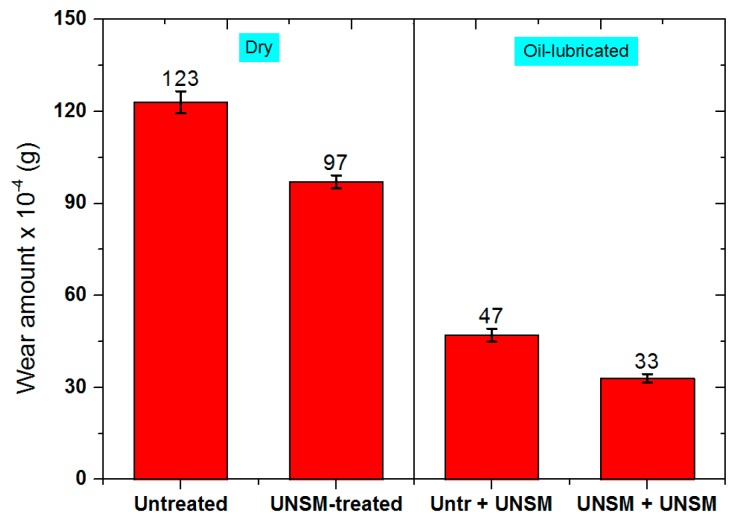
Comparison of wear amount for the untreated and UNSM-treated Babbitt specimens in dry and oil-lubricated conditions, respectively.

A comparison of the extent of the wear of the specimens after a sliding time of 60 min in dry and oil-lubricated conditions is shown in Figure 9. The extent of wear in the untreated and UNSM-treated specimens was found to be 22.3 mg and 15.2 mg, and 12.2 mg and 9.7 mg under dry and oil-lubricated conditions, respectively. Kobernik et al. introduced a method of producing the Babbitt coating by plasma arc surfacing [25]. It was found that the wear resistance of the deposited Babbitt coating was higher than that of cast Babbitt alloy, but the friction coefficient was not reduced. Moreover, Barykin et al. investigated the influence of grain size of β-phase on wear resistance of Babbitt alloy [26]. According to the wear test results, it was found that the wear resistance was increased with decreasing β-phase grain size. 

**Figure 10:**
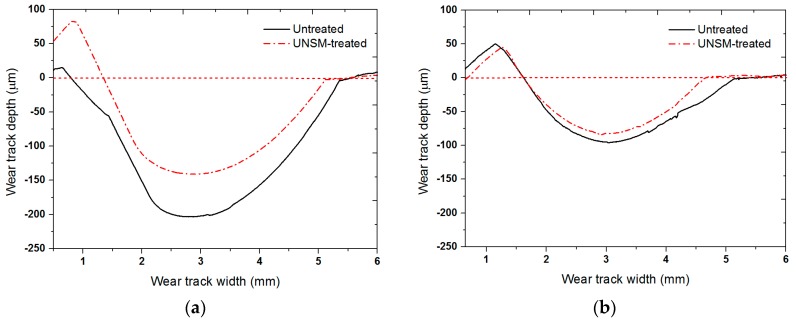
Comparison of cross-sectional wear track profiles of the untreated and UNSM-treated Babbitt specimens in (**a**) dry and (**b**) oil-lubricated conditions, respectively.

Figure 10 shows the cross-sectional profile of the wear tracks on the surface of the untreated and UNSM-treated specimens formed under dry and oil-lubricated conditions. The width and depth of the wear tracks on the untreated specimens were 4.62 and 3.85 µm, and 204 and 140 µm, and on the UNSM-treated specimens, they were 3.51 and 3.02 µm, and 96 and 81 µm, under dry and oil-lubricated conditions, respectively. As a result, it was confirmed that the UNSM technique effectively increased the resistance of Babbitt alloy to wear in both dry and oil-lubricated conditions.

**Figure 11:**
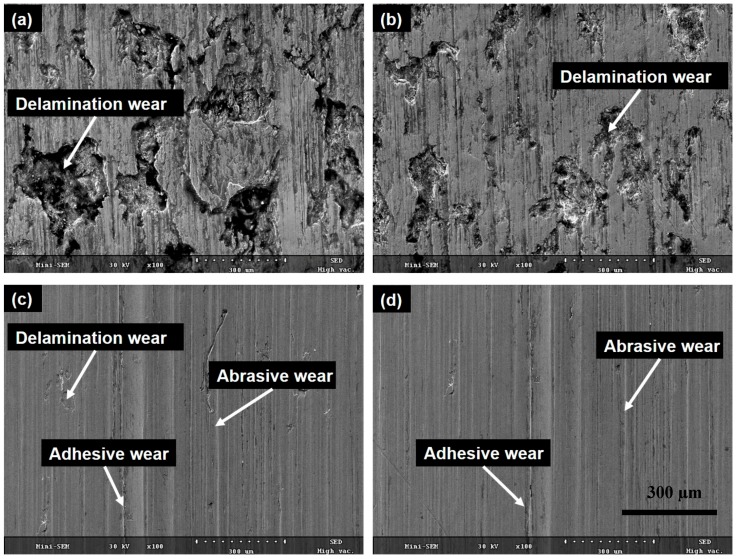
Wear track scanning electron microscopy (SEM) images of the Babbitt specimens in dry and oil-lubricated conditions. (**a**) Untreated in dry conditions; (**b**) UNSM-treated in dry conditions; (**c**) untreated in oil-lubricated conditions and (**d**) UNSM-treated in oil-lubricated conditions.

**Figure 12:**
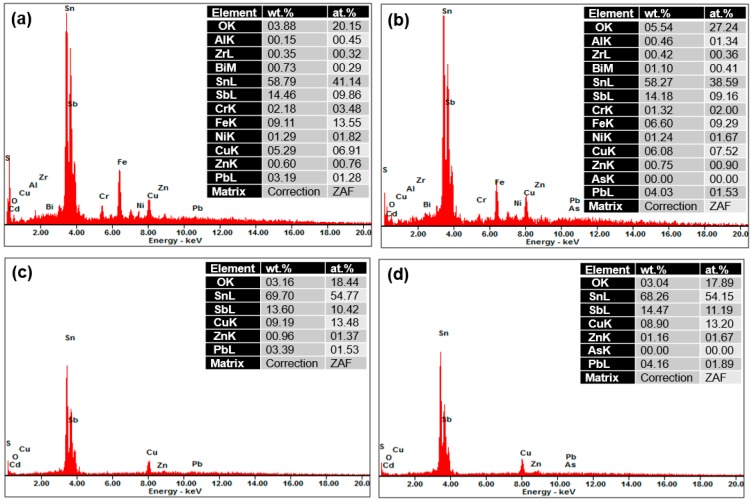
Energy-dispersive X-ray spectroscopy (EDX) results obtained from the wear track of the Babbitt specimens in dry and oil-lubricated conditions. (**a**) Untreated in dry conditions; (**b**) UNSM-treated in dry conditions; (**c**) untreated in oil-lubricated conditions and (**d**) UNSM-treated in oil-lubricated conditions.

SEM images of wear tracks formed on the untreated and UNSM-treated specimens in dry and oil-lubricated conditions are shown in Figure 11a,b. It was observed that the untreated specimens exhibited a severe wear compared to the UNSM-treated specimens under both dry and oil-lubricated conditions. The specimens showed a similar morphology on the worn-out surface under dry conditions, indicating a delamination wear mechanism with grooves parallel to the sliding direction (see Figure 11a), with the delamination wear being relatively mild for the UNSM-treated specimen. The wear mechanism of the specimens under oil-lubricated conditions was found to be abrasive and adhesive in nature, as shown in Figure 11c,d. In addition, delamination wear was also observed for the untreated specimen under oil-lubricated conditions. Moreover, it can be seen that the inner surface of the wear track in the UNSM-treated specimen—under both dry and oil-lubricated conditions—was found to be smoother compared to the untreated specimen; this is known as the smoothing phenomenon [27]. It is of interest to analyze the worn-out surfaces by energy-dispersive X-ray spectroscopy (EDX), as shown in Figure 12, to elucidate the possible wear mechanism and to understand the material transfer during sliding. It was found that the oxidation wear occurred in almost identical amounts of O on the specimens under dry conditions, but under oil-lubricated conditions, more O was detected on the UNSM-treated specimen. However, the worn-out surfaces under dry conditions showed more elements (Fe, Zr, etc.), while no such elements were found on the worn surface of the specimens under oil-lubricated conditions. This confirms that less material transfer occurred under oil-lubricated conditions than under dry conditions. It has been previously reported that the O generated at the contact interface can reduce the friction coefficient and the extent of wear [28]. The improvement in the friction and wear behavior of Babbitt material may be attributed to the increased hardness, reduced surface roughness, and the parallel grooves produced by the UNSM technique. As a result, it is expected that the application of the UNSM technique to the Babbitt metal of journal bearings of a cone ore crusher should be adopted by the mining industry, in order to save energy and increase the efficiency and productivity of ore cone crushers.

## 4. Conclusions

In this study, the effects of the UNSM technique on the surface roughness, surface hardness, friction, and wear behavior of Babbitt alloy were investigated. It was found that the surface roughness was reduced and the surface hardness increased after UNSM treatment. The friction and wear behavior improved due to the increase in hardness and reduction in surface roughness. Thus, it is expected that the application of the UNSM technique to Babbitt alloys can increase the efficiency of the ore cone crusher. Further friction and wear tests with respect to load and rotating speed under both dry and oil-lubricated conditions need to be performed.

## Figures and Tables

**Table 1:** Chemical composition of Babbitt specimens (in wt.%).

Sn	Sb	Cu	Impurities					
			**Pb**	**Fe**	**Al**	**Bi**	**As**	**Zn**
Balance	8.5	5	0.4	0.08	0.01	0.08	0.1	0.01

**Table 2:** Optimized ultrasonic nanocrystalline surface modification (UNSM) treatment parameters for Babbitt specimens.

Frequency (kHz)	Amplitude (µm)	Static Load (N)	Speed (rpm)	Interval (µm)	Ball (mm)
20	30	2	20	70	6 (Si_3_N_4_)
